# MYC-type transcription factors, MYC67 and MYC70, interact with ICE1 and negatively regulate cold tolerance in Arabidopsis

**DOI:** 10.1038/s41598-018-29722-x

**Published:** 2018-08-02

**Authors:** Masaru Ohta, Aiko Sato, Na Renhu, Tsuyoshi Yamamoto, Nodoka Oka, Jian-Kang Zhu, Yasuomi Tada, Takuya Suzaki, Kenji Miura

**Affiliations:** 1 Graduate School of Life and Environmental Sciences, University of Tsukuba, Tsukuba, 305-8572 Japan; 2 Graduate School of Science, Nagoya University, Nagoya, 464-8602 Japan; 3 Department of Horticulture and Landscape Architecture, Purdue University, West Lafayette, IN 47906 USA; 4 Shanghai Center for Plant Stress Biology and Center of Excellence in Molecular Plant Sciences, Chinese Academy of Sciences, Shanghai, 200032 China

## Abstract

The expression of hundreds of genes is induced by low temperatures via a cold signaling pathway. ICE1, a MYC-type transcription factor, plays an important role in the induction of *CBF3*/*DREB1A* to control cold-responsive genes and cold tolerance. To elucidate other molecular factors, a yeast 2-hybrid screening was performed. Two MYC-type transcription factors, MYC67 and MYC70, were identified as ICE1-interacting proteins. The *myc* mutants were more tolerant to freezing temperatures than wild type. *CBF3*/*DREB1A* and other cold-responsive genes were up-regulated in the *myc* mutants. Overexpression of the *MYC* genes increased the cold sensitivity and down-regulated the expression of cold-responsive genes. The MYC proteins interacted with the *cis-*elements in the *CBF3*/*DREB1A* promoter, probably to interfere interaction between ICE1 and the *cis-*elements. Taken together, these results demonstrate that MYC67 and MYC70, ICE1 interactors, negatively regulate cold-responsive genes and cold tolerance.

## Introduction

Plants are continuously exposed to various stresses in their natural environment. To acclimate to several kinds of stress conditions at the molecular, cellular, and at the whole plant levels, plants have evolved intricate mechanisms.

Plant growth and development are highly affected by these environmental stresses, such as cold, drought, and salt stresses. Although many tropical plants are cold-sensitive and significant losses of yield are observed under freezing temperatures, most temperate plants have ability to tolerate to freezing temperatures following a period of exposure to non-freezing temperatures. This process is known as cold acclimation. The expression of many genes is induced by the cold signaling pathway during acclimation to cold stresses. These genes include those encoding the following: anti-freezing proteins; molecular chaperones; proteins for respiration; proteins involved in metabolism of carbohydrates, lipids, and antioxidants; and factors that putatively function in freezing-induced dehydration tolerance^1^. C-repeat (CRT)/dehydration responsive element (DRE) *cis*-elements are found in the promoters of many cold- and dehydration-responsive genes that encode proteins helping plants to respond to cold^2,3^. The CBF (CRT-binding factor)/DREB1 (DRE-binding protein) proteins are a family of AP2-type transcription factors, which binds to the core sequence CCGAC of CRT/DRE *cis*-elements to induce expression of cold- and dehydration-responsive genes^4,5^. Because the expression of CBF/DREB1 factors is also induced by cold temperatures^4,5^, other transcription factors are required for the induction of *CBF*/*DREB1* expression in response to cold stress.

Several factors involved in the regulation of *CBF*/*DREB* expression have been genetically identified in Arabidopsis^1^. The *ice1* mutation was identified using a genetic screen for mutants, which down-regulated expression of *CBF3:LUC* reporter gene^6^. The expression of *CBF3*/*DREB1A* and its downstream target genes was down-regulated in the *ice1* mutant; accordingly, the *ice1* plants significantly exhibited chilling and freezing sensitive^6^. ICE1, MYC-like bHLH (basic-helix-loop-helix) transcription factor, recognizes the canonical MYC *cis*-elements (CANNTG) in the *CBF3*/*DREB1A* promoter^6^ to regulate expression of *CBF3*/*DREB1A*. A genome-wide transcript profiling demonstrated that the *ice1* mutation affected the expression levels of 204 genes among 939 cold-regulated genes^7^. On the other hand, the expression of *CBF1*/*DREB1B*, not *CBF3*/*DREB1A*, was primarily influenced by ICE2, a homolog of ICE1^8^. Therefore, ICE1 and ICE2 play important roles in the transcriptional regulation of *CBF*/*DREB1* genes.

Understanding the function of ICE1 and its regulation of cold-responsive genes would contribute to elucidation of ICE1-dependent cold response pathway. In the present study, we performed a yeast 2-hybrid screen and focused on two ICE1-interacting factors, MYC67 and MYC70. Because the *myc6*7 or *myc*7*0* mutant exhibited cold tolerance and *MYC67-* or *MYC70-*overexpression lines exhibited cold sensitive, these factors play negative role in regulation of cold tolerance. The MYC67 and MYC70 were able to bind to the *MYC cis-*elements in the promoter of *CBF3*/*DREB1A*. The ChIP assay revealed that binding activity of ICE1 to the *MYC cis-*elements was increased in the *myc67* or *myc70* mutants. Increased binding activity of ICE1 may enhance cold responsive gene transcription and tolerance. These results suggest that MYC67 or MYC70 inhibits interaction between ICE1 and *MYC cis-*elements, leading to negative regulation of cold tolerance.

## Results

### Identification of MYC67 and MYC70 as ICE1-interacting proteins

**Figure 1:**
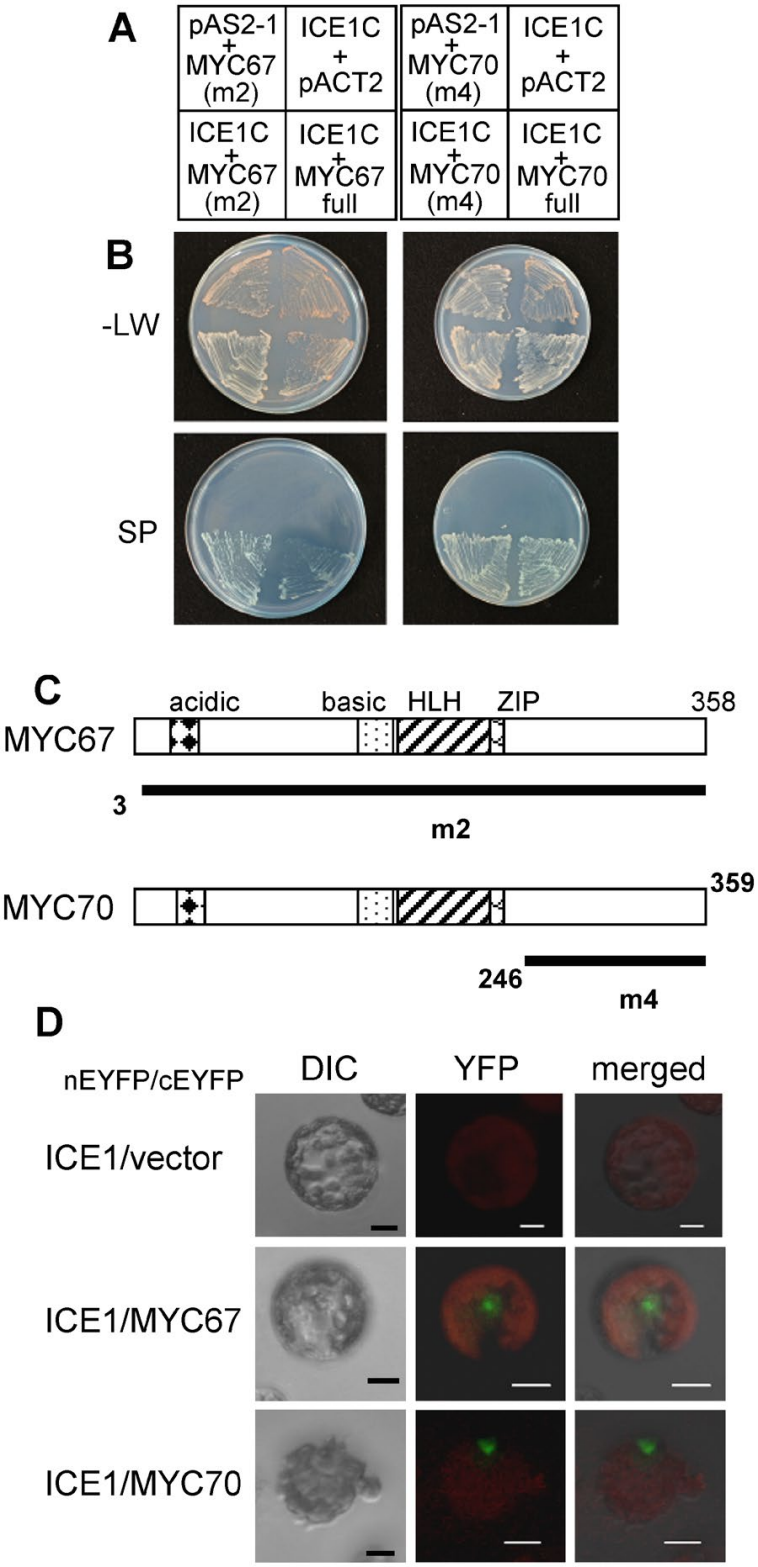
MYC67 and MYC70 interact with ICE1. Yeast 2-hybrid analysis. (**A**) A schematic representation of the bait (top) and prey (bottom) combinations. The yeast strains contained pAS2-ICE1-C (amino acids 267–494) as bait together with pACT-MYC67 m2 (amino acids 3–358), pACT2-MYC67 full (amino acids 1–358), pACT2-MYC70 m4 (amino acids 246–359), or pACT2-MYC70 full (amino acids1–359) as prey. pAS2-1 and pACT2 were used as negative controls. (**B**) The yeast strains were grown on synthetic dropout (SD) plates without leucine and tryptophan (−LW) or on selection plates (SP), which consisted of an SD plate containing X-α-gal and lacking leucine, tryptophan, histidine and adenine. (**C**) The domain structures of MYC67 and MYC70. Acidic, basic, helix-loop-helix (HLH), ZIP domains are presented. Black bars indicate the region of MYC67 m2 (amino acids 3–358) or MYC70 m4 (amino acids 246–359), respectively. (**D**) BiFC analysis indicated interaction between ICE1 and the MYC proteins. ICE1 was fused to N-terminal EYFP (enhanced yellow fluorescent protein), and the MYC proteins were fused to C-terminal EYFP. Each combination of constructs was transfected into Arabidopsis protoplasts. Differential interference contrast (DIC) images and the YFP fluorescence from the BiFC analysis are shown along with the merged image. The scale bar indicates 10 μm.

To identify molecular factors that regulate cold signaling, a yeast 2-hybrid screen was performed using ICE1 as the bait. Because full-length ICE1 and N-terminal region of ICE1 activated the transcription of a GAL4-responsive reporter gene in yeast, we used the C-terminal region of ICE1 (ICE1-C, amino acid residues 267–494) as the bait. Three types of prey libraries from ABRC were screened^9–11^, and 38 clones were confirmed to positively express *HIS3*, *ADE2*, and *MEL1*. A sequence analysis of the prey plasmids revealed that four of the encoded proteins were bHLH-type transcription factors. One of these clones encodes FAMA (At3g24140), which was previously reported to interact with ICE1^12^. The other clones encode bHLH transcription factors, MYC67 and MYC70, which belong to the same subfamily as FAMA^13^. m2 and m3 encode MYC67 (amino acids 3–358 of At3g61950), and m4 encodes MYC70 (amino acids 246–359 of At2g46810) (Fig. 1A–C).

To confirm the interactions of full-length MYC67 and MYC70 with ICE1-C, the cDNAs were amplified by RT-PCR. The full-length coding region of MYC67 was amplified from a cDNA library that was derived from 10-day-old seedlings, but MYC70 was not amplified from the same cDNA library. To reevaluate the gene structure, the genomic sequence of MYC70 was analyzed using Eukaryotic GeneMark (http://opal.biology.gatech.edu/GeneMark/eukhmm.cgi). The open reading frame predicted by Eukaryotic GeneMark did not match the sequence of At2g46810.1 (Fig. S1A). The predicted coding region of MYC70 (At2g46810.3) was amplified with a pair of primers specific to the predicted ORF, indicating that the predicted ORF was transcribed *in vivo*. Hereafter, we used At2g46810.3 (AB678434.1 in GenBank database) as the MYC70 full-length ORF (Fig. S1B). The full-length MYC67 and MYC70 proteins were interacted with ICE1-C (Fig. 1B). By using At2g46810.3 for phylogenic analysis (Fig. S2), MYC67 and MYC70 have similar amino acid sequences.

To assess *in vivo* interactions between ICE1 and the MYC proteins, a bimolecular fluorescence complementation (BiFC) assay was performed^14^. ICE1 was fused to the N-terminal region of enhanced yellow fluorescence protein (nEYFP), and the MYC proteins were fused to the C-terminal region of EYFP (cEYFP). These constructs were transformed into Arabidopsis mesophyll protoplasts and transiently expressed. Fluorescence at nuclei was detected when ICE1 was co-expressed with MYC67 or MYC70 but not done when ICE1 was co-expressed with a vector (Fig. 1D). These results indicate that ICE1 can interact with these MYC proteins *in vivo*.

**Figure 2:**
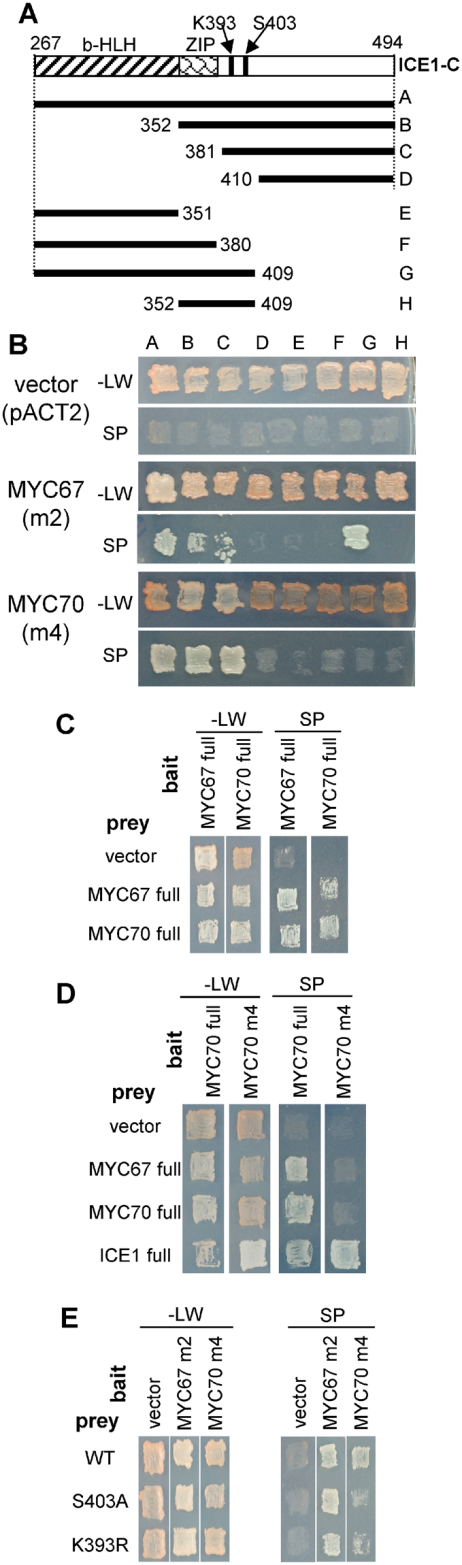
Interactions between ICE1, MYC67, and MYC70. (**A**) A schematic representation of the deletion constructs for ICE1. The indicated regions of ICE1 were cloned into the bait vector pGBKT7. (**B**) The interaction of ICE1 with MYC67 or MYC70 was assessed using the yeast 2-hybrid assay. The ICE1 bait plasmid was transformed with either pACT2 (vector control), pACT2-MYC67 m2 (amino acids 3–358), or pACT2-MYC70 m4 (amino acids 246–359). Yeast grown on SD media without leucine and tryptophan (upper, −LW) and on selection plates (lower, SP) are shown. (**C**) MYC67 and MYC70 form homodimers and interact with each other. Yeast strains containing full-length pGBKT7-MYC67 or pGBKT7-MYC70 as bait were transformed with pACT2 (vector control), full-length pACT2-MYC67 or full-length pACT2-MYC70 as prey. Yeast was incubated on −LW or on SP. (**D**) Only ICE1 was able to interact with the C-terminal region of MYC70 in the yeast 2-hybrid assay. MYC70 full was interacted with MYC67, MYC70, and ICE1, but MYC70 m4 was able to interact with ICE1. (**E**) The effect of ICE1 point mutations, S403A or K393R, on the interactions with MYC67 and MYC70.

To determine which region of ICE1 interacts with the MYC proteins, several regions of ICE1 were used in a yeast 2-hybrid assay (Fig. 2A). MYC67 m2 and MYC70 m4 were able to interact with region C (amino acids 381–494) of ICE1 (Fig. 2B), suggesting that bHLH region of ICE1 is not required for the interaction of these MYC proteins. Because MYC67 m2 also interacted with region G, the region for interaction with MYC67 was determined to be amino acids 381–409 of ICE1. On the other hand, interaction with MYC70 m4 required the C-terminal region of ICE1 (Fig. 2B).

The MYC proteins were also found to form homodimers and interact with each other (Fig. 2C). Although the C-terminal portion (amino acids 246–359, m4) of MYC70, which lacks the bHLH domain, was unable to interact with full-length MYC67 or full-length MYC70 (Fig. 2D). On the other hand, MYC70 m4 interacts with ICE1 (Fig. 2D). This result suggests that homodimer of MYC70 and heterodimer formation between MYC67 and MYC70 requires bHLH domain of MYC70.

Several key amino acid residues are present in the C-terminal region of ICE1, including K393 and S403, which are required for the sumoylation and stabilization of the protein, respectively^15,16^. Because the C-terminal region of ICE1 was found to be necessary for interaction with MYC67 and MYC70 (Fig. 2B), the interaction of ICE1(K393R) and ICE1(S403A) with MYC67 and MYC70 was examined. These mutated versions of ICE1 were able to interact with MYC67 and MYC70 (Fig. 2E), suggesting that the mutations do not affect interaction.

### *MYC67* and *MYC70* negatively regulate cold tolerance

The homozygous T-DNA insertion lines SALK_009770 and SALK_069605 for *MYC67* and *MYC70*, respectively, were isolated (Fig. S3A). Semi-quantitative RT-PCR revealed that transcripts for the respective *MYC* genes were not detected in these *myc* mutants (Fig. S3B). The growth of *myc67* or *myc70* plants was similar to that of wild-type plants under normal conditions. *MYC67-* or *MYC70-*overexpressing lines were also generated for further functional analysis (Fig. S3C).

**Figure 3:**
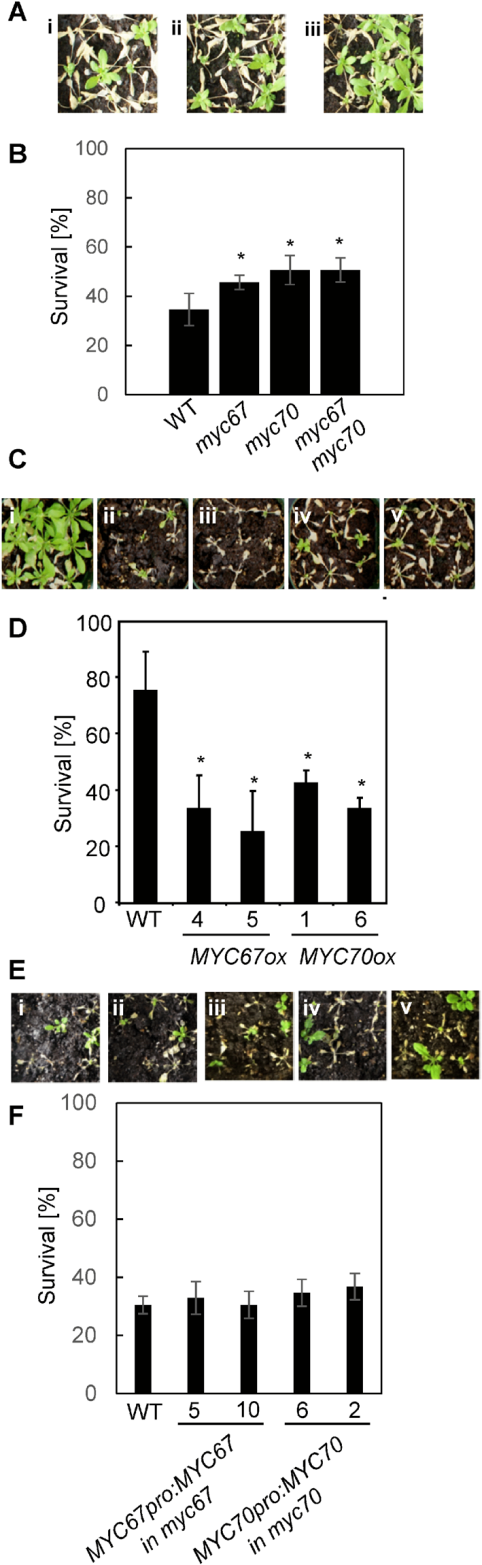
*MYC67* and *MYC70* negatively regulate freezing tolerance. (**A**) The freezing tolerance of *myc67* and *myc70* after cold acclimation. Representative wild-type (i), *myc67* (ii) and *myc70* (iii) plants that were treated with −7 °C are shown. (**B**) Quantification of the survival rates for the cold-acclimated wild-type, *myc67*, and *myc70* plants after a −7 °C freezing treatment. The data indicate the means and standard errors (n = 6). The tolerance of *myc67*, *myc70*, and *myc67 myc70* mutants was significantly different from that of wild-type plants (t-test, *P* < 0.05). (**C**) *MYC67*-, and *MYC70*-overexpressing plants exhibited freezing sensitivity. The plants shown are representative wild-type plants (i), *MYC67-*overexpressing plants #4 (ii) and #5 (iii), and *MYC70-*overexpressing plants #1 (iv) and #6 (v). (**D**) Quantification of the survival rates for the cold-acclimated *MYC67*-, and *MYC70*-overexpressing plants after a −6 °C freezing treatment. The data indicate the means and standard errors (n = 6). The survival ratios of *MYC67*-, and *MYC70*-overexpressing plants were significantly different than that of wild-type plants (t-test, *P* < 0.05). (**E**) *MYC67pro:: FLAG:MYC67* or *MYC70pro:: FLAG:MYC70* was expressed in *myc67* or *myc70* mutant, respectively. The mutant phenotype was complemented. The plants shown are representative wild-type (i), *myc67* plants with *MYC67pro::FLAG:MYC67* #2-1 (ii) and #2-4 (iii), and *myc70* plants with *MYC70pro:: FLAG:MYC70* #3-1 (iv) and #5-2 (v). (**F**) Quantification of the survival rates for the cold-acclimated *myc67* plants with *MYC67pro::FLAG:MYC67* and *myc70* plants with *MYC70pro:: FLAG:MYC70* after freezing treatment at −6 °C. The data indicate the means and standard errors (n = 6).

Three-week-old wild-type and *myc* plants were subjected to −7 °C for 4 hr after cold acclimation. After a one-week recovery period at room temperature, the mutant plants exhibited a higher survival rate than the wild-type plants (Fig. 3A,B). Because MYC67 and MYC70 have similar amino acid sequences (Fig. S2), the double mutant was generated. The *myc67 myc70* double mutant exhibited similar phenotype as did each mutant (Fig. 3B). On the other hand, *MYC*-overexpression transgenic plants that were subjected to −6 °C for 4 hr after cold acclimation were more sensitive to freezing temperature than wild-type plants (Fig. 3C,D). These results indicate that *MYC67* and *MYC70* negatively regulate cold tolerance. To confirm complementation of the *myc67* or *myc70* mutation with *MYC67* or *MYC70*, respectively, *MYC67pro::FLAG:MYC67* in *myc67* or *MYC70pro::FLAG:MYC70* in *myc70* was generated (Fig. S3). The plants with similar expression level of *MYC67* or *MYC70*, compared to that in wild type, was used for freezing assay (Fig. 3E,F), demonstrating that each *MYC* gene was able to complement each mutation.

**Figure 4:**
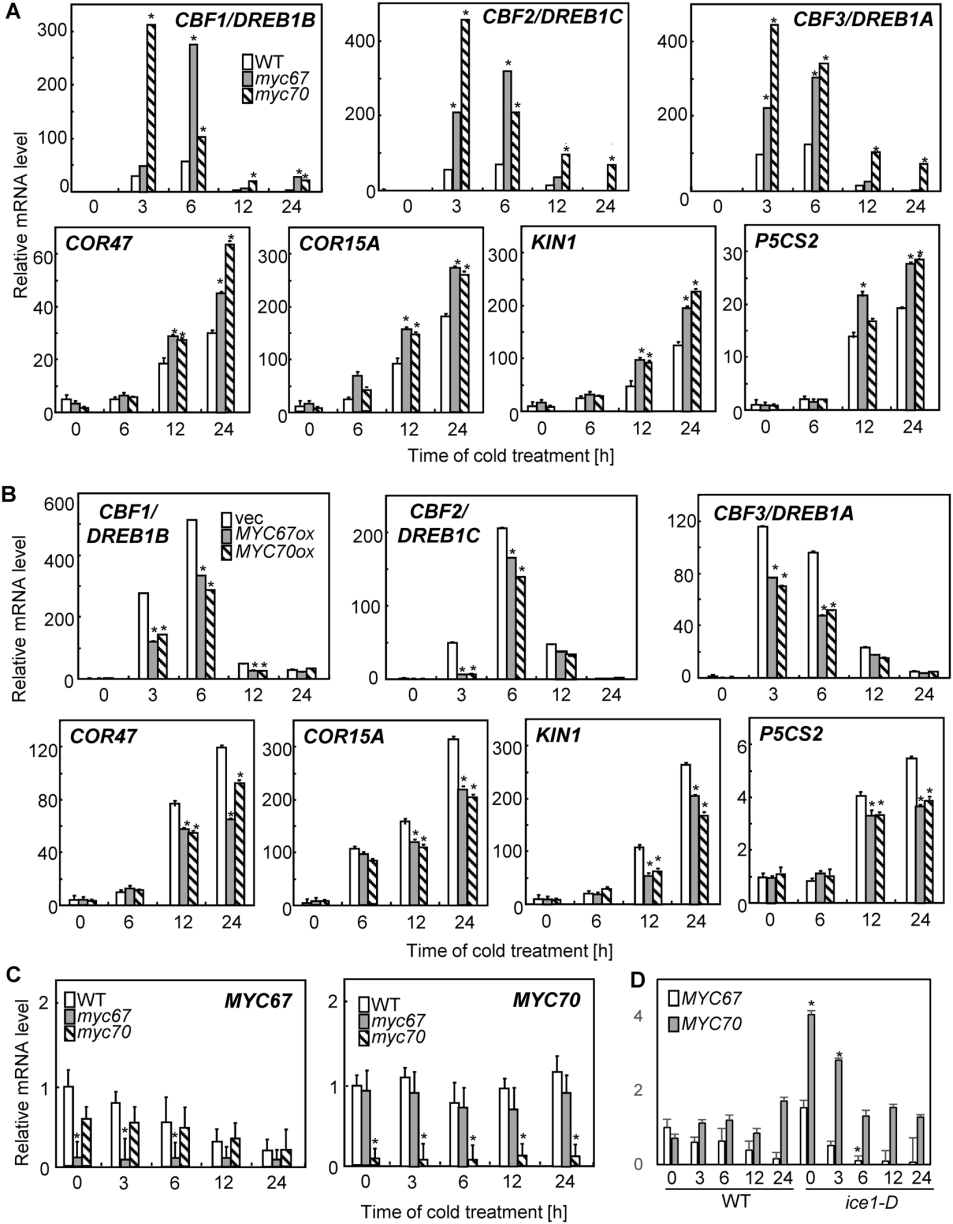
*MYC67* and *MYC70* negatively regulate the expression of cold-responsive genes. (**A**) The relative mRNA transcript levels of *CBF*/*DREB1* genes, *COR47*, *COR15A*, *KIN1*, and *P5CS2* in wild-type, *myc67* and *myc70* seedlings were determined by quantitative RT-PCR analysis. Ten-day-old seedlings grown at 23 °C were incubated at 4 °C for the indicated amounts of time. The data indicate the means and SD (n = 3). (**B**) The relative mRNA transcript levels in wild-type, *MYC67*-, and *MYC70*-overexpressing seedlings were determined by quantitative RT-PCR. The data indicate the means and SD (n = 3). (**C**) The expression levels of *MYC67* and *MYC70* after cold treatment were measured by quantitative RT-PCR analysis. (**D**) Expression level of *MYC67* and *MYC70* after cold treatment in wild type and the *ice1*-*D* mutant. Asterisks indicates a significant difference from wild-type or plants with a vector at each point (p < 0.05) as determined by Student’s *t-*tests.

Several cold-responsive genes are ICE1-target genes^7^. Thus, quantitative RT-PCR analysis was performed. The cold-induced transcript accumulation of *CBF*/*DREB1* genes and their dependent cold-responsive genes, including *COR47*, *COR15A*, *KIN1*, and *P5CS2*, was increased in the *myc67* and *myc70* mutants (Fig. 4A). Additionally, the expression of these genes was reduced in *MYC67-* and *MYC70-*overexpression lines (Fig. 4B), suggesting that these *MYC* genes act as negative regulators of cold signaling. The expression of *MYC67* was decreased after cold treatment, whereas expression level of *MYC70* was not significantly altered in the presence or absence of cold treatment (Fig. 4C). Each mutation did not affect expression of another *MYC* gene. In the *ice1-D* mutant, *MYC70* was highly expressed in normal condition and was decreased after cold treatment (Fig. 4D). Expression level of *MYC70* was increased in the *ice1-D* mutant.

Because ICE1 is involved in stomatal development^12^, the stomata on the abaxial surfaces of rosette leaves of the *myc67* or *myc70* were observed. The mature guard cells of the *myc* mutants were indistinguishable from wild-type guard cells (Fig. S4).

### Expression and localization of MYC67 and MYC70

**Figure 5:**
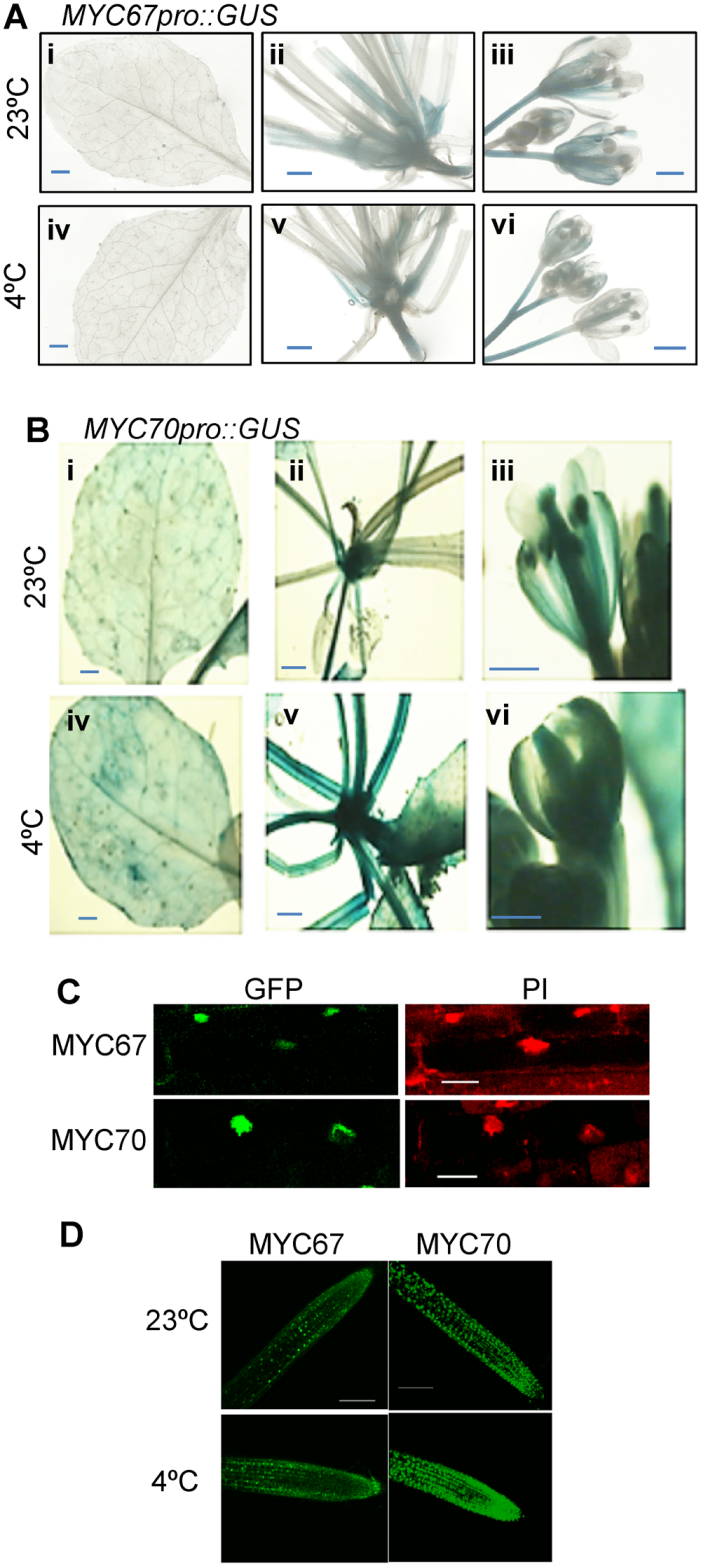
Expression and localization of *MYC67* and *MYC70*. (**A**) Six-week-old plants expressing *MYC67pro:GUS* was treated without (i–iii) or with (iv–vi) low temperature for 6 h. *MYC67pro:GUS* expression in mature rosetta leaves (i,iv), base of rosetta leaves (ii, v), and floral organs (iii,vi). (**B**) Six-week-old plants expressing *MYC70pro:GUS* was treated without (i–iii) or with (iv–vi) low temperature for 6 h. *MYC70pro:GUS* expression in mature rosetta leaves (i, iv), base of rosetta leaves (ii,v), and floral organs (iii, vi). (**C**) Subcellular localization of the GFP-MYC proteins. The nuclei were stained with 20 mg/ml PI. The scale bar indicates 20 μm. (**D**) GFP fluorescence at the root tips. Seven-day old seedlings were treated at 4 °C for 3 h, and GFP fluorescence was observed by confocal microscopy. The scale bar indicates 150 μm.

The expression pattern of each *MYC* gene was analyzed using promoter:GUS fusion transgenes. Six-week-old plants harboring *MYC67pro:GUS* or *MYC70pro:GUS* was treated with or without low temperature. Then, *MYC67pro:GUS* or *MYC70pro:GUS* expression was observed in base of rosetta leaves and floral organs (Fig. 5A,B). In mature leaves, only *MYC70pro::GUS* expression was observed (Fig. 5B). Then, expression pattern was compared after cold treatment. Before or after cold treatment, expression pattern of *MYC67pro::GUS* and *MYC70pro::GUS* was not altered (Fig. 5A,B). Expression level of *MYC70pro::GUS* was slightly increased, probably because mRNA level of *MYC70* was not altered under cold treatment (Fig. 4C) and GUS protein may be accumulated after cold treatment. Even though no signal of *MYC67pro::GUS* was observed in 6-week-old rosetta leaves, the expression was observed around wound sites with normal condition (Fig. S5). It is suggested that expression of *MYC67* is response to wound.

To evaluate the localization of the MYC proteins, transgenic plants expressing GFP-MYC fusion proteins under the control of the constitutive CaMV 35 S promoter were prepared. GFP fluorescence was observed in the nuclei, indicating that MYC67 and MYC70 proteins are localized to the nucleus (Fig. 5B). All GFP-MYC transgenic plants were subjected to low-temperature conditions (4 °C) for 3 h to assess the effects on GFP emission. The intensities of GFP-MYC67 and GFP-MYC70 fluorescence were similar to those in non-treated plants (Fig. 5C).

### MYC67 and MYC70 bind to *MYC cis*-elements in the *CBF3*/*DREB1A* promoter

There are five possible *MYC*-recognition elements in the *CBF3*/*DREB1A* promoter, and ICE1 interacts specifically with these *MYC cis-*elements^6^. Because MYC67 and MYC70 have bHLH domains, it is likely that these MYC proteins recognize *MYC cis-*elements.

**Figure 6:**
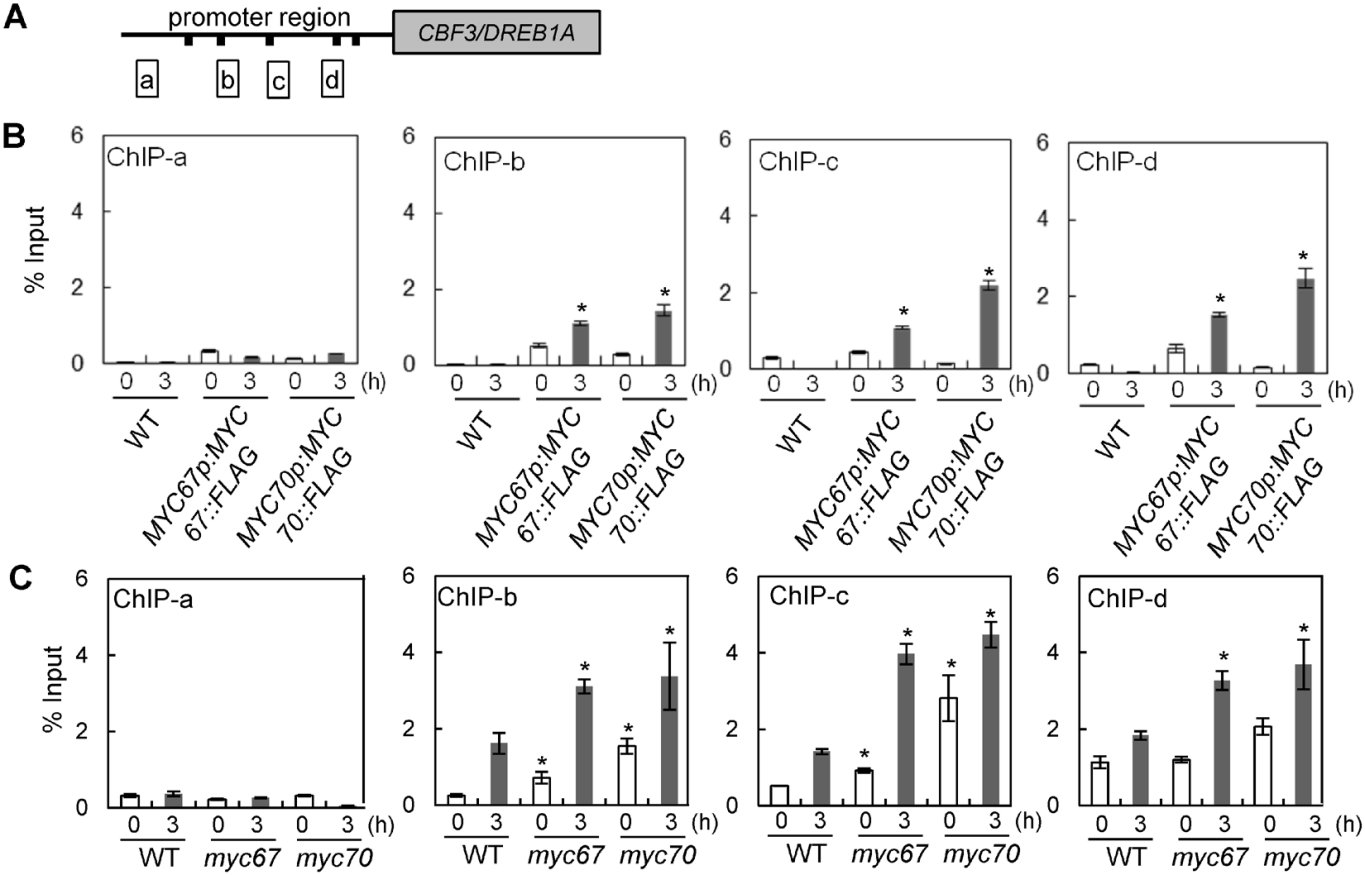
MYC67 and MYC70 were able to bind to the promoter region of *CBF3*/*DREB1A*. (**A**) A schematic representation of the promoter region of *CBF3*/*DREB1A* and the region that was amplified after the ChIP analysis. (**B**) The wild-type and transgenic plants used for the ChIP assay were treated at 4 °C for 3 h. The immunoprecipitated DNA with anti-DYKDDDDK tag monoclonal antibody was quantified by quantitative PCR using primers in the promoter region. The data are the average of technical triplicates for the quantitative PCR (mean ± SD). (**C**) The wild-type and *myc* mutants were treated at 4 °C for 3 h, and ChIP analysis was performed with anti-ICE1 antibody.

To investigate the binding activity of MYC proteins at the promoter of *CBF3*/*DREB1A in vivo*, a chromatin immunoprecipitation (ChIP) assay was performed using an anti-DYKDDDDK tag monoclonal antibody and the immunoprecipitated DNA was amplified (Fig. 6A,B). After cold treatment, the binding activity of MYC67 and MYC70 to the *CBF3*/*DREB1A* promoter was increased (Fig. 6B). It is probably because negative regulators are also important in cells for fine tuning. When ChIP assay was performed using an anti-ICE1 antibody in the wild-type or *myc* mutant, the binding activity of ICE1 to the *CBF3*/*DREB1A* promoter was increased in the *myc67* or *myc70* mutant (Fig. 6C). Because no canonical *MYC cis-*element in the region a, no significant binding activity was detected (Fig. 6B,C). These results suggest that the binding activity of ICE1 is suppressed by MYC67 or MYC70, probably leading to suppression of some downstream cold responsive genes and tolerance.

## Discussion

In the present study, we isolated bHLH-type transcription factors, MYC67 and MYC70, that function as ICE1 interactors. T-DNA insertions in these genes improved cold tolerance and enhanced the expression of cold-regulated genes. Furthermore, the overexpression of *MYC67* and *MYC70* decreased cold tolerance and down-regulated cold-regulated genes. These results indicate that *MYC67* and *MYC70* act as negative regulators of cold signaling and tolerance in Arabidopsis. Additionally, these MYC proteins recognize the *MYC cis-*elements in the promoters of *CBF*/*DREB1* genes. The binding activity of ICE1 to the promoter of *CBF3*/*DREB1A* may be suppressed by MYC67 and MYC70, leading to reduction of cold tolerance.

The MYC proteins were able to bind to the *MYC cis-*elements in the promoters of *CBF*/*DREB1* genes (Fig. 6), which were previously shown to be bound by ICE1^6^. These MYC proteins were also able to form homodimers and heterodimers (Fig. 2C). The heterodimerization of bHLH proteins can either stimulate or inhibit transcription. Previous characterization of the Myc/Max/Mad network has shown that Max can form heterodimers with either Myc or Mad in vertebrates. The Myc-Max heterodimer acts as a transcriptional activator, whereas the Mad-Max heterodimer acts as a transcriptional repressor^17^. Another reason may be the heterodimer of MYC67 and MYC70 cover *MYC cis-*elements to inhibit binding of ICE1 to *MYC cis*-elements. Because the *myc67 myc70* double mutant exhibited similar cold tolerance as did each mutant (Fig. 3B), it is suggested that hetero-dimerization of MYC67 and MYC70 may be required for suppression of cold tolerance. According to ChIP analysis, binding activity of ICE1 to *MYC cis-*elements was increased in cold-treated plants and in the *myc67* or *myc70* mutant (Fig. 6C). Together, these cold-induced changes of binding activity may contribute to the induction of cold signaling and cold acclimation and MYC67 and MYC70 may inhibit activity of ICE1 to suppress cold signaling and tolerance.

We previously identified K393 and S403 as important residues in ICE1^15,16^. K393 is a target for sumoylation that is mediated by the SUMO E3 ligase SIZ1 and the sumoylation of ICE1 is important for the inhibition of ubiquitylation and cold tolerance^15^. The substitution of S403 for alanine in ICE1 enhances the following: the stabilization of ICE1; the expression of cold-regulated genes, including *CBF3*/*DREB1A*, *COR47*, and *KIN1*; and cold tolerance^16^. The interaction between ICE1 and MYC67 or MYC70 was observed (Fig. 2E), suggesting that the interaction may be unaffected by posttranslational modification. Although ICE1 primarily affects the expression of *CBF3*/*DREB1A*^6^, ICE2 primarily influences the expression of *CBF1*/*DREB1B* and not *CBF3*/*DREB1A*^8^. The increased expression of *CBF1*/*DREB1B* in the *myc67* or *myc70* mutant (Fig. 4A) suggest that MYC67 and MYC70 may interact with ICE2 or affect its activity. Among 1256 cold-induced genes, 11% of these genes were upregulated by overexpression of *CBF*/*DREB1*^18^. The low-temperature regulatory network is highly interconnected by CBF/DREB1 and other transcription factors^18^. Regulation of other transcription factors by ICE1, MYC67, or MYC70 is to be elucidated.

Phosphorylation of ICE1 at S278 by OST1/SnRK2.6 kinase enhances stability of ICE1 through interfering with the interaction between HOS1 and ICE1^19^. High-density protein microarray analysis identified 570 phosphorylation substrates of MPK (MAP kinase)^20^. According to these data, ICE1 is phosphorylated by MPK5 and MPK6. In the previous study, MKK2 (MAP kinase kinase 2) phosphorylates MPK4 and MPK6 in response to cold and the overexpression of a constitutively active MKK2 in plants confers cold and salt stress tolerance^21^. These results suggest that phosphorylation of ICE1 activates its transactivation activity. On the other hand, neither MYC67 nor MYC70 was found to be a substrate of MPK in the protein array^20^, suggesting that regulatory mechanism may be different.

In summary, MYC67 and MYC70 interact with ICE1 and act as negative regulators of cold signaling. Because the ICE1-dependent cold signaling cascade is conserved in several plants, such as tomato^22,23^, rice^24^, and wheat^25^, similar MYC interacting proteins may regulate cold tolerance through the expression of *CBF*/*DREB* and cold-regulated genes in various species.

## Methods

### Yeast 2-hybrid assay

The C-terminal region of ICE1 (amino acids 267–494) was amplified from pMAL-ICE1^6^ with 5a-1 and MAL-3 primers. The PCR product was digested with *Sma*I and *Sal*I, cloned into *Sma*I and *Sal*I sites of the pAS2 vector (Table S1). pAS2-ICE1-C was transformed into the AH109 *Saccharomyces cerevisiae* yeast strain (Matchmaker GAL4 Two-Hybrid System 3, Clontech) and incubated on selective medium lacking tryptophan (SD-W). cDNA libraries (CD4-10, CD4-22, CD4-30) were obtained from the Arabidopsis Biological Resource Center and then transformed into the AH109 yeast strain containing pAS2-ICE1-C, which was then incubated on SD lacking tryptophan, leucine, histidine, and adenine (SD-WLHA). Colonies were transferred to SD-WLHA with 20 μg/ml of X-α-Gal (5-bromo-4-chloro-3-indolyl α-D-galactopyranoside) to confirm the expression of the *MEL1* reporter gene, which is positive for α-galactosidase activity. The prey vector was obtained from yeast and transformed into yeast containing pAS2-ICE1-C or pAS2 (the vector control); the transformed yeast was then incubated on SD-WLHA containing X-α-Gal. Prey vectors were sequenced when the yeast harboring the prey vector and pAS2-ICE1-C survived and the yeast with the prey vector and pAS2 did not.

The full-length coding regions of the MYC67 and MYC70 prey vectors were constructed as follows. At2g46810.3 was amplified by PCR using primers MYC70-4 and MYC70-5 (Table S1). The coding regions of MYC67 and MYC70 were amplified by PCR using gene-specific primers (MYC67-1, MYC67-2, MYC70-7, and MYC70-2; Table S1), digested with *Xma*I and *Sal*I, and introduced into *Xma*I- and *Xho*I-digested pACT2 or into *Xma*I- and *Sal*I*-*digested pGBKT7. The plasmids of the bait and prey constructs were transformed into the yeast AH109 strain. The positive colony was obtained from SD-WLHA with X-α-Gal.

To construct the ICE1-deletion clones (A-H) and ICE1-mutant clones (K393R and S403A), portions of wild-type ICE1, ICE1(K393R) and ICE1(S403A) were amplified by PCR using specific primers (Table S1), digested with *Eco*RI and *Bam*HI, and introduced into pGBKT7 at the corresponding restriction sites.

### BiFC assay

The coding region of *ICE1* was amplified using primers AtICE1-17 and AtICE1-3 (Table S1). The PCR products were digested with *Eco*RI and *Bam*HI and then inserted into the *Eco*RI and *Bam*HI sites of the pSAT6-nEYFP-C1 vector^14^. To make the *cEYFP-MYC* plasmids, the coding regions of *MYC67* and *MYC70* were amplified with primers MYC67-7, MYC70-11, and NOS-transR (Table S1). The resultant PCR products were digested with *Eco*RI and *Sal*I and inserted into the *Eco*RI and *Sal*I sites of the pSAT6-cEYFP-C1 vector^14^. The constructs were transiently expressed in Arabidopsis leaf protoplasts, which were prepared from two-week-old wild-type seedlings using Cellulase Ononzuka R-10 and Macerozyme R-10 (Yakult Pharmaceutical) as previously described^26^. The plasmid DNA of nEYFP-ICE1 and cEYFP-MYC was introduced into Arabidopsis protoplasts by polyethylene glycol-mediated transfection^27^. After an overnight incubation at 22 °C, the fluorescent and differential interference contrast (DIC) images were observed with a microscope (IRBE-4, Leica).

### Plant materials and growth conditions

The Arabidopsis T-DNA insertion mutants *myc67-1* (SALK_009770) and *myc70-1* (SALK_069605) were obtained from the Arabidopsis Biological Resource Center at Ohio State University. T-DNA insertion was confirmed by genotyping with primers SALK009770L, SALK009770R, SALK069605L, SALK069605R, and Salk_LBa1 (Table S3).

The T7 tag was introduced into the CaMV35S-Nos vector by inverse PCR using primers T7-1 and T7-2 (Table S1). The resultant CaMV35S-T7tag-Nos vector was digested with *Hin*dIII and *Eco*RI, and the fragment was introduced into the plant binary vector pCAMBIA1300 to generate pCAMH-35S-T7tag-Nos. The coding regions of MYC67 and MYC70 were excised from pGBKT7-MYC67 and pGBKT7-MYC70, respectively, by digestion with *Xma*I and *Sal*I. The resultant fragments were introduced into the *Xma*I and *Xho*I sites of pCAMH-35S-T7tag-Nos. pCAMH-35S-T7-MYC67 and pCAMH-35S-T7-MYC70 were transformed into Arabidopsis (Col-0 or the respective mutant for complementation) by *Agrobacterium* (strain GV3101)-mediated transformation^16^. The transgenic lines were selected on 1/2 MS agar plates with 30 μg/mL hygromycin. The transgenic plants were selected, and quantitative RT-PCR was performed using gene-specific forward primers (MYC67-5 and MYC70-10) and NOS-transR (Table S2) to determine the expression levels of each transgene (Fig. 3E).

The whole plant freezing assay was performed as previously described^16^. Cold-acclimated plants were treated at −6 °C or −7 °C for 4 hr in a programmed incubator (IN602, Yamato, Japan). After the freezing treatment, the plants were stored overnight at 4 °C and then transferred to 23 °C. The survival ratio was determined one week after the freezing treatment.

### RNA preparation and quantitative RT-PCR

Wild-type, *myc* mutant, and *MYC*-overexpressing plants were grown on 1/2 MS agar plates at 23 °C for 10 d under a long-day photoperiod (16 h light/8 h dark). The plants were then subjected to cold treatments at 4 °C. Total RNA was isolated using TRIZOL reagent (Invitrogen) according to the manufacturer’s instructions^16^. cDNA was synthesized from 2 μg of total RNA using the High Capacity cDNA Reverse Transcription Kit (Applied Biosystems, USA). The primers used for real-time PCR are listed in Table S2. Real-time PCR amplification and detection were carried out using SYBR Premix ExTaq (Takara Bio, Japan) on a Thermal Cycler Dice TP800 (Takara Bio, Japan). The relative transcript abundance was calculated using the comparative C_T_ method as previously described^15^, with *Actin2*/*7* as the control standard. Values representative the means ± SD (n = 3) from representative experiments from three or more independent experiments.

### Microscopic Analysis

The coding region of GFP was amplified with the primers GFPN-1 and GFPN-2 (See Supplemental Table S1). The resultant PCR product was inserted into the *Sma*I site of the CaMV35S-Nos vector to make CaMV35S-GFPN-Nos. The plasmid DNA with *GFP* in the sense direction was digested with *Hin*dIII and *Eco*RI, and the fragment was introduced into the plant binary vector pCAMBIA1300 to make pCAMH-35S-GFPN-Nos. The coding regions of MYC67 and MYC70 were excised from pGBKT7-MYC67 and pGBKT7-MYC70, respectively, by digestion with *Xma*I and *Sal*I. The fragments were then introduced into the *Xma*I and *Xho*I sites of pCAMH-35S-GFPN-Nos.

The GFP-MYC67 and GFP-MYC70 plasmids were introduced into *Agrobacterium* and transformed into wild-type *Arabidopsis* plants (Col-0). T2 transgenic lines that were resistant to hygromycin were chosen for the analysis of GFP expression. The fluorescence of the GFP-MYC fusion proteins was observed with a confocal laser-scanning microscope (TCS SP2, Leica).

The promoter region of *MYC67* (1086 bp upstream from the initiation codon) was amplified using gene-specific primers (MYC67pro-pCAMBIA1391Z-F and MYC67pro-pCAMBIA1391Z-R; Table S1). The PCR product was digested with *Eco*RI and *Nco*I and were inserted into pCAMBIA1391Z with In-Fusion HD Cloning Kit (Takara Bio, Japan) to generate MYC67pro-GUS. The promoter region of *MYC70* (1474 bp upstream from the initiation codon) was amplified using gene-specific primers (MYC70.2pro-pCAMBIA1391Z-F and MYC70.2pro-pCAMBIA1391Z-R; Table S1). The PCR products was inserted into *Eco*RI and *Nco*I and were inserted into pCAMBIA1391Z to generate MYC70pro-GUS. GUS staining was performed as previously described^28^. Representative seedlings were photographed using a microscope (IRBE-4, Leica).

The DIC images were used for the quantitative and qualitative examination of the epidermal phenotypes. Seven-day-old seedlings were fixed in 1:9 (v:v) acetic acid:ethanol overnight, rinsed with water and then placed in chloral hydrate (1:8:1 glycerol:chloral hydrate:water v:w:v). The samples were visualized using a microscope (IRBE-4, Leica). To score the stomatal number, the number of cells in 0.25 mm^2^ regions of wild-type and mutant leaves were counted, and the means were calculated.

### Chromatin Immunoprecipitation (ChIP) Assay

To investigate whether MYC proteins were interacted with the promoter region of *CBF*s, ChIP assay was performed. Transgenic plants expressing *MYC67pro::FLAG:MYC67* or *MYC70pro::FLAG:MYC70* were generated. *FLAG* fused with *MYC67 or MYC70* was amplified with the primers, pRI201-3xFLAG-F, 3xFLAG-R, 3xFLAG-MYC-F, and MYC-pRI201-R (Table S1), then PCR products were introduced into pRI201-AN (Takara Bio, Japan) digested with *Nde*I and *Sal*I with In-Fusion HD Cloning Kit (Takara Bio, Japan). After construction, 35 S promoter was exchanged with *MYC67pro* or *MYC70pro*, which was amplified with the primers, pRI201-MYC67pro-F and pRI201-MYC67pro-R, or pRI201-MYC70.2pro-F and pRI201-MYC70.2pro-R, respectively. The DNA region of *MYC67pro::FLAG:MYC67:HSPterminator* or *MYC70pro::FLAG:MYC70:HSPterminator* was amplified with the primers pCsV-MYC67pro-F and pCsV-HSPter-R, or pCsV-MYC70.2pro-F and pCsV-HSPter-R, respectively, and the PCR products were introduced into *Pvu*II digested pCsV1300 with In-Fusion HD Cloning Kit. The constructs were designated as MYC67pro::FLAG:MYC67-CsV and MYC70.2pro::FLAG:MYC70.2-CsV, which were transformed into *myc67* or *myc70* mutants, respectively. Expression level of *FLAG:MYC67* or *FLAG:MYC70* was examined by RT-PCR with the primers 3xFLAG-F and 3xFLAG-R (Fig. S3).

Two-week-old seedlings were treated with or without cold (4 °C) for 3 h, and the seedlings were subsequently harvested and cross-linked with buffer A (0.4 M sucrose; 10 mM Tris-HCl, pH 8; 1 mM EDTA; 1 mM PMSF; 1% formaldehyde). The cross-linking reaction was stopped with 125 mM glycine. The tissues were ground with liquid nitrogen, resuspended with lysis buffer (50 mM HEPES, pH 7.5; 150 mM NaCl; 1 mM EDTA; 1% Triton X-100; 0.1% deoxycholate; 0.1% SDS; 1 mM PMSF; and 1X protease inhibitor cocktail II), and sonicated (Microson XL-2000 sonicator, Qsonica, LLC., USA) to achieve an average fragment size of 0.1 to 1.0 kb. After sonication, centrifugation was performed, and the supernatants were incubated overnight at 4 °C with anti-DYKDDDDK tag monoclonal antibody (Wako Pure Chemical Industies, Japan) or anti-ICE1 rabbit polyclonal antibody generated from peptide CQEDGSSQFKPMLEG (Scrum Inc., Japan). The immunoprecipitation was performed using Magna ChIP A Chromatin Immunoprecipitation Kits (Millipore).

Quantitative PCR was performed with THUNDERBIRD SYBR Premix (Toyobo) using a real-time PCR Thermal Cycler Dice (Takara) as previously described^16^. The relative quantities of immunoprecipitated DNA fragments were calculated as the percentage of input chromatin that was immunoprecipitated using the comparative C_T_ method. The primer sequences are listed in Supplemental Table S4.

### Accession numbers

Sequence data from this article can be found in the GenBank/EMBL databases under the following accession number: *MYC70*, At2g46810.3 (AB678434.1). Other sequence data can be found in the Arabidopsis Genome Initiative database under the following accession numbers: *ACT2*/*7*, At5g09810 (NP_196543); *ICE1*, At3g26744 (NP_001030774); *MYC67*, At3g61950 (NP_001325697).

## Electronic supplementary material

Supplementary Information
