# Supplementary material for: MYC-type transcription factors, MYC67 and MYC70, interact with ICE1 and negatively regulate cold tolerance in Arabidopsis
Source: Sci Rep. 2018 Aug 2;8:11622. doi: 10.1038/s41598-018-29722-x (PMC6072781; doi:10.1038/s41598-018-29722-x)
Supplement: Supplementary file 1 — Supplementary Information [file 41598_2018_29722_MOESM1_ESM.pdf]

Supplementary Information for *Scientific Reports*

MYC-type transcription factors, MYC67 and MYC70, interact with ICE1 and negatively regulate cold tolerance in Arabidopsis

Masaru Ohta[1], Aiko Sato[1], Na Renhu[1], Tsuyoshi Yamamoto[1], Nodoka Oka[2],
Jian-Kang Zhu[3, 4], Yasuomi Tada[2], Takuya Suzaki[1], Kenji Miura[1,*]

[1]Graduate School of Life and Environmental Sciences, University of Tsukuba, Tsukuba 305-8572, Japan

[2]Graduate School of Science, Nagoya University, Nagoya 464-8602, Japan

[3]Department of Horticulture and Landscape Architecture, Purdue University, West Lafayette, IN 47906, USA
[4]Shanghai Center for Plant Stress Biology and Center of Excellence in Molecular Plant Sciences, Chinese Academy of Sciences, Shanghai 200032, China

A

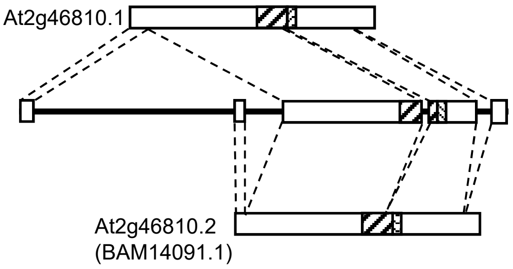

B

```
   1  ATGGAGAGGAGTATTCAAGGACAAAACAAGCTCTGTTGTTTGGACCAAAAAGTGAATGTGAGAAGAAGCCTACAAGTTCAAGAAACTGTA    90
      M  E  R  S  I  Q  G  Q  N  K  L  C  C  L  D  Q  K  V  N  V  R  R  S  L  Q  V  Q  E  T  V

  91  GAGGATCATCAAAGCTTTGCCCTTGAAGAGGAAGAACAACAACTCTCAACTCCGAGCTTGCTGCAAGACACAACAATACCATTTCTACAA   180
      E  D  H  Q  S  F  A  L  E  E  E  E  Q  Q  L  S  T  P  S  L  L  Q  D  T  T  I  P  F  L  Q

 181  ATGCTGCAACAAAGTGAAGACCCTTCACCGTTTTTGTCATTCAAAGACCCAAGCTTTCTAGCACTACTATCTCTCCAGACACTTGAAAAG   270
      M  L  Q  Q  S  E  D  P  S  P  F  L  S  F  K  D  P  S  F  L  A  L  L  S  L  Q  T  L  E  K

 271  CCTTGGGAACTCGAAAACTACCTCCCACATGAAGTTCCAGAGTTTCATTCACCGATCCATTCTGAAACCAACCACTACTATCATAATCCA   360
      P  W  E  L  E  N  Y  L  P  H  E  V  P  E  F  H  S  P  I  H  S  E  T  N  H  Y  Y  H  N  P

 361  TCTTTGGAAGGAGTCAATGAAGCCATCTCAAACCAAGAACTTCCATTCAACCCACTAGAGAATGCGCGTTCAAGACGCAAGCGGAAAAAC   450
      S  L  E  G  V  N  E  A  I  S  N  Q  E  L  P  F  N  P  L  E  N  A  R  S  R  R  K  R  K  N

 451  AACAACTTGGCATCATTGATGACAAGAGAAAAGCGAAAGAGAAGAAGAACTAAACCAACAAAGAACATAGAAGAGATAGAGAGTCAAAGA   540
      N  N  L  A  S  L  M  T  R  E  K  R  K  R  R  R  T  K  P  T  K  N  I  E  E  I  E  S  Q  R

 541  ATGACACACATTGCGGTTGAACGAAACCGCAGACGCCAAATGAACGTTCATCTGAACTCACTCCGCTCCATCATTCCATCTTCATACATC   630
      M  T  H  I  A  V  E  R  N  R  R  R  Q  M  N  V  H  L  N  S  L  R  S  I  I  P  S  S  Y  I

 631  CAGAGGGGAGACCAAGCGTCAATAGTAGGAGGAGCAATAGACTTCGTAAAGATCCTAGAGCAACAGTTGCAATCCCTTGAAGCACAAAAG   720
      Q  R  G  D  Q  A  S  I  V  G  G  A  I  D  F  V  K  I  L  E  Q  Q  L  Q  S  L  E  A  Q  K

 721  AGAAGTCAACAGAGTGATGATAACAAAGAGCAAATTCCAGAAGATAACAGTCTCAGGAACATTTCGTCGAACAAGTTGCGTGCGAGTAAT   810
      R  S  Q  Q  S  D  D  N  K  E  Q  I  P  E  D  N  S  L  R  N  I  S  S  N  K  L  R  A  S  N

 811  AAAGAAGAACAAAGTAGCAAACTCAAAATCGAAGCCACAGTGATAGAGAGTCACGTCAACCTAAAAATTCAATGTACGAGGAAACAAGGA   900
      K  E  E  Q  S  S  K  L  K  I  E  A  T  V  I  E  S  H  V  N  L  K  I  Q  C  T  R  K  Q  G

 901  CAACTTCTCAGATCAATCATATTGCTGGAGAAACTTCGATTCACTGTTCTTCATCTCAACATCACATCTCCGACCAATACATCTGTCTCT   990
      Q  L  L  R  S  I  I  L  L  E  K  L  R  F  T  V  L  H  L  N  I  T  S  P  T  N  T  S  V  S

 991  TATTCCTTCAACCTCAAGATGGAAGATGAATGTAATTTGGGATCAGCGGATGAGATAACGGCGGCGATTCGTCAGATTTTCGACAGCTGA  1080
      Y  S  F  N  L  K  M  E  D  E  C  N  L  G  S  A  D  E  I  T  A  A  I  R  Q  I  F  D  S  *
```

Figure S1. The nucleotide and amino acid sequence of At2g46810.2/MYC70, which was newly identified in this study. (A) The exon/intron structure of *MYC70*. The boxes indicate the exons. The structures of the mature transcripts, At2g46810.1 and At2g46810.2 are shown. (B) The nucleotide and amino acid sequence of At2g46810.2/MYC70. The sequence was registered as AB678434.1 in GenBank database.

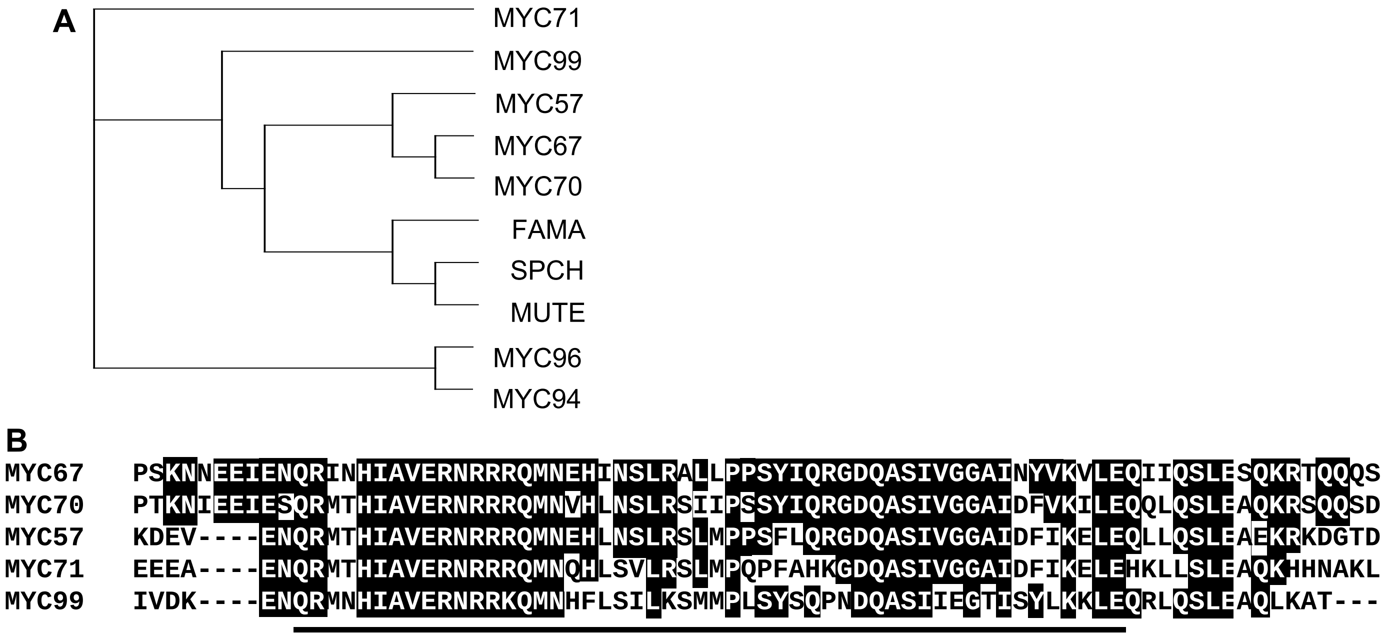

Figure S2. Molecular phylogeny of bHLH proteins in subfamily 3 (A). A phylogenetic analysis of the full-length amino acid sequences of subfamily 3 bHLH (Toledo-Ortiz et al. 2003) was performed using the default settings of CLUSTALW ver. 1.83 in DDBJ (http://clustalw.ddbj.nig.ac.jp/top-j.html). The amino acid sequence of At2g46810.3 (newly identified in this study, Figure S2) was used as the MYC70 sequence for this phylogenic analysis. (B) Sequence alignment of the bHLH domains (underline) and its flanking regions of MYC67, MYC70, and other bHLH proteins. Identical residues with MYC67 are shown in black.

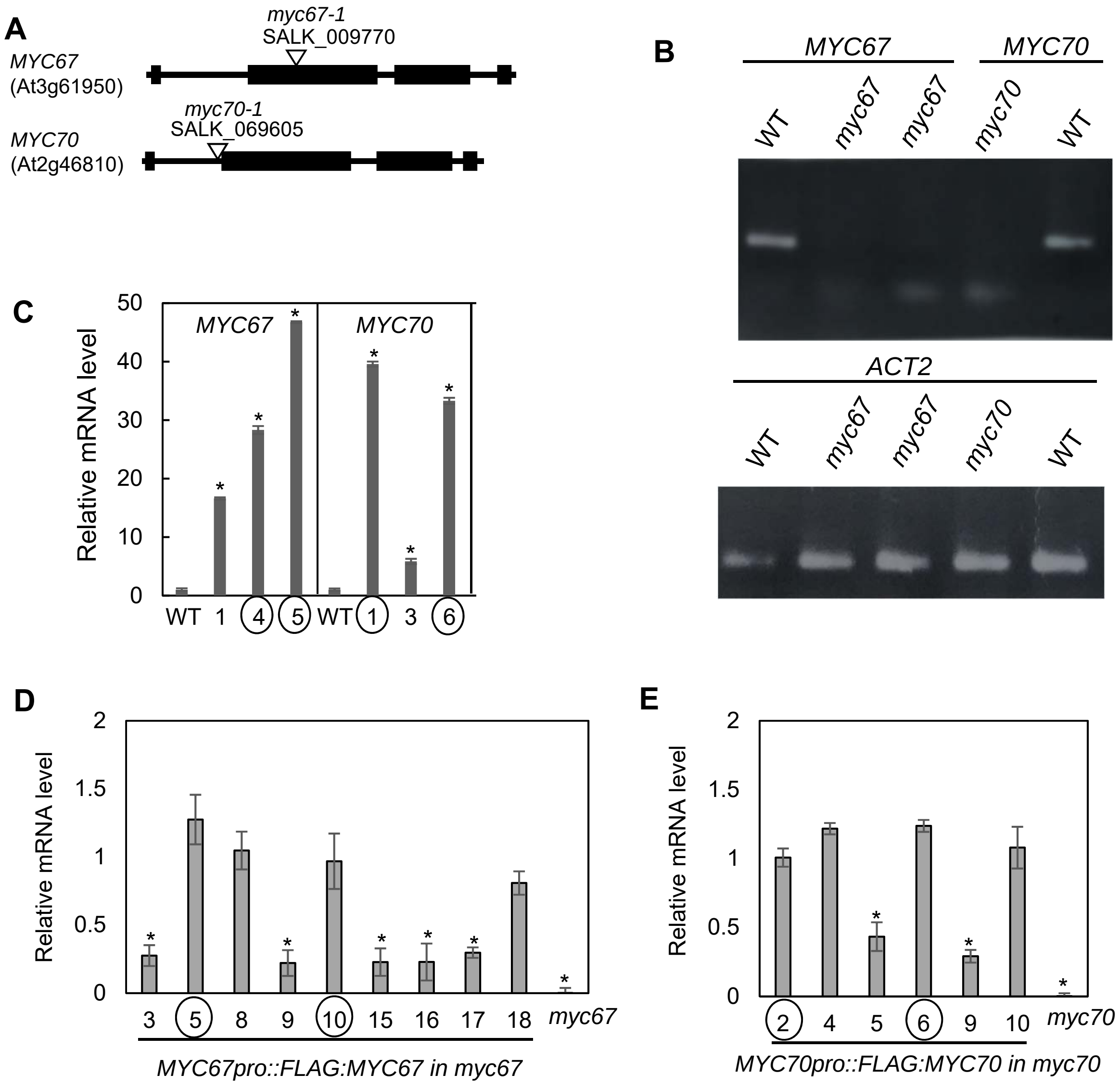

Figure S3. Expression level of the *myc* mutants and *MYC*-overexpression lines. (A) Schematic diagrams of the *myc67* and *myc70*. (B) The expression of *MYC67* or *MYC70* was not observed in the *myc67* or *myc70* mutant, respectively. For control, *ACT2* expression was detected in the wild type, *myc67,* and *myc70* mutant (bottom panel). M, DNA marker. (C) Expression of *MYC67* or *MYC70* was determined by quantitative PCR method. The lines 4 and 5 of *MYC67*-overexpression lines and lines 1 and 6 of *MYC70*-overexpression lines were used for further experiments, respectively. (D, E) The expression of *MYC67* (D) or *MYC70* (E) in the *MYCpro::FLAG:MYC* transgenic lines was determined. The expression level of *MYC67* or *MYC70* in wild type was set as 1. The circled line was used for complementation assay and ChIP analyses. Asterisks indicates a significant difference from wild-type or plants with circles (D and E) as determined by Student's *t*-tests ($p < 0.05$) .

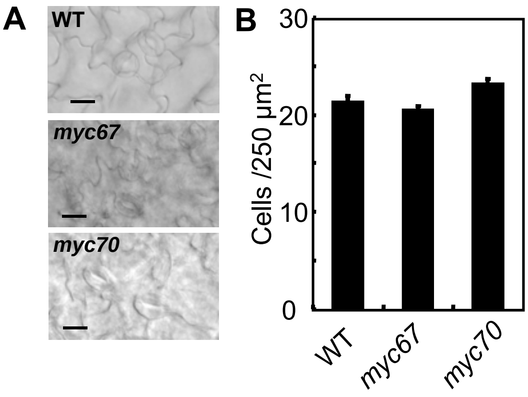

Figure S4. Stomatal development in the *myc67* and *myc70* mutants. (A) The abaxial leaf epidermis of wild-type, *myc67* and *myc70* plants. The scale bar indicates 10 µm. (B) The mean number of stomata on the abaxial leaf epidermis. Data are presented as means ± SE (n = 30).

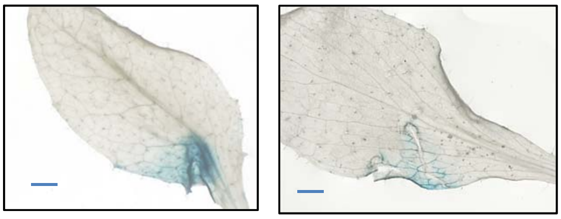

Figure S5. *MYC67pro:GUS* expression was observed around wound sites in 6-week-old rosetta leaves.

Table S1. Primers used for plasmid construction.

Name of primer	DNA sequence (5'-to-3')
**pAS2-ICE1-C**	
5a1-10	TGAGACTGGGATTGAGGTTTCTG
MAL-3	CAAGCTTGCCTGCAGGTCGAC
**pGEM-MYC70 (for isolation of At2g46810.2)**	
MYC70-4	GTTACCAAAGTAAACGTTTC
MYC70-5	GATGGAGAGGAGTATTCAAGG
**pACT2-MYC67 & pGBKT7-MYC67**	
MYC67-1	GACCCCGGGTATGGAAAGGTTTCAAGGACA
MYC67-2	TTTTGGAATTATATGTGTCGACTCAAATTG
**pACT2-MYC70 & pGBKT7-MYC70**	
MYC70-7	GACCCCGGGTATGGAGAGGAGTATTCAAG
MYC70-2	TACTTTTTACTTGGATTGTCGACTCAGCTG
**CaMV35S-T7-Nos**	
T7-1	CACCAGTCATGCTAGCCATCTGTAATTGTA
T7-2	GACAGCAAATGGGTCGCCCGGGGGTACCGT
**CaMV35S-GFPN-Nos**	
GFPN-1	CATGGTGAGCAAGGGCGAGGAGC
GFPN-2	GGGGCCTTGTACAGCTCGTCCATGC
**MYC67-GUS**	
MYC67pro-pCAMBIA1391Z-F	GGATCCCCGGGAATTCAATAAGTTGTCTTGTCTTGAAAAAGG
MYC67pro-pCAMBIA1391Z-R	CTCAGATCTACCATGGTTGAGAGCTCTTGTTTGGTATGTAATG
**MYC70-GUS**	
MYC70.2pro-pCAMBIA1391Z-F	GGATCCCCGGGAATTCGTCACGATACAAATCGAGCGCTAAAAG
MYC70.2pro-pCAMBIA1391Z-R	CTCAGATCTACCATGGCAGAAAGCTGAGCTCTTTTTCTTTC
**nEYFP-ICE1**	
AtICE1-17	CGGAATTCTATGGGTCTTGACGGAAAC
AtICE1-3	GCGGATCCTCAGATCATACCAGCATAC
**cEYFP-MYC67 & MYC70**	
MYC67-7	CGGAATTCTATGGAAAGGTTTCAAGGAC
MYC70-11	CGGAATTCTATGGAGAGGAGTATTCAAG
NOS-transR	GCCAAATGTTTGAACGATCGGGAA

Table S1. (continued)

Name of primer	DNA sequence (5'-to-3')
**MYC67pro::FLAG:MYC67-CsV or MYC70.2pro::FLAG:MYC70.2-CsV**	
pRI201-3xFLAG-F	CACTGTTGATACATATGATCGATACCGTCGAGATGGACTAC
3xFLAG-R	AATTCGAGCTCTGTCGACTGTGG
3xFLAG-MYC67-F	GACAGAGCTCGAATTATGGAAAGGTTTCAAGGACACATC
MYC67-pRI201-R	ATTCAGAATTGTCGACTCAAATTGTCGGAATATCGAAAATC
3xFLAG-MYC70-F	GACAGAGCTCGAATTATGGAGAGGAGTATTCAAGGACAAAAC
MYC70-pRI201-R	ATTCAGAATTGTCGACTCAGCTGTCGAAAATCTGACGAATC
pRI201-MYC67pro-F	CCAAGCTTGCATGCCAATAAGTTGTCTTGTCTTGAAAAAG
pRI201-MYC67pro-R	ACGGTATCGATCATATTGAGAGCTCTTGTTTGGTATGTAATG
pRI201-MYC70.2pro-F	CCAAGCTTGCATGCCGTCACGATACAAATCGAGCGCTAAAAG
pRI201-MYC70.2pro-R	ACGGTATCGATCATACAGAAAGCTGAGCTCTTTTTCTTTC
pCsV-MYC67pro-F	TCGCTATTACGCCAGAATAAGTTGTCTTGTCTTGAAAAAGG
pCsV-MYC70.2pro-F	TCGCTATTACGCCAGGTCACGATACAAATCGAGCGCTAAAAG
pCsV-HSPter-R	GATTCATTAATGCAGCTTATCTTTAATCATATTCCATAGTC

Table S1. (continued)

Name of primer	DNA sequence (5'-to-3')
**ICE1 deletion**	
**Region A (267/494)**	
AtICE1-1	GCGAATTCACTGGGATTGAGGTTTCTGGG
AtICE1-3	GCGGATCCTCAGATCATACCAGCATAC
**Region B (352/494)**	
AtICE1-2	GCGAATTCCTTCTACAAAGGATCAATG
AtICE1-3	GCGGATCCTCAGATCATACCAGCATAC
**Region C (381/494)**	
AtICE1-4	GCGAATTCTTGACACCTACACCGCAAAC
AtICE1-3	GCGGATCCTCAGATCATACCAGCATAC
**Region D (410/494)**	
AtICE1-5	GCGAATTCAGAGTTGAGGTTAGATTAAG
AtICE1-3	GCGGATCCTCAGATCATACCAGCATAC
**Region E (267/351)**	
AtICE1-1	GCGAATTCACTGGGATTGAGGTTTCTGGG
AtICE1-6	GCGGATCCTATTCCTTCAGATAATCAATTGC
**Region F (267/380)**	
AtICE1-1	GCGAATTCACTGGGATTGAGGTTTCTGGG
AtICE1-7	GCGGATCCTACGGATGGAAGCTTGATG
**Region G (267/409)**	
AtICE1-1	GCGAATTCACTGGGATTGAGGTTTCTGGG
AtICE1-8	GCGGATCCTAAGCTTGCTGGCCTTTAGGAC
**Region H (352/409)**	
AtICE1-2	GCGAATTCCTTCTACAAAGGATCAATG
AtICE1-8	GCGGATCCTAAGCTTGCTGGCCTTTAGGAC

Table S2. Primers used for detection of cold-responsive genes

Name of primer	DNA sequence (5'-to-3')
CBF1/DREB1B	GCATGTCTCAACTTCGCTGA
	ATCGTCTCCTCCATGTCCAG
CBF2/DREB1C	TGACGTGTCCTTATGGAGCTA
	CTGCACTCAAAAACATTTGCA
CBF3/ DREB1A	GATGACGACGTATCGTTATGGA
	TACACTCGTTTCTCAGTTTTACAAAC
COR15A	GCTTCAGATTTCGTGACGGATAAAAC
	GCAAAACATTAAAGAATGTGACGGTG
COR47	CAGTGTCGGAGAGTGTGGTG
	ACAGCTGGTGAATCCTCTGC
KIN1	TGGAGCTGGAGCACAACA
	GACCCGAATCGCTACTTGTTC
P5CS2	GACAGTGCTGCTGTTTTCCA
	AACTTGTCCCTTTCCTCTCATTATC
ACTIN2	TAACAGGGAGAAGATGACTCAGATCA
	AAGATCAAGACGAAGGATAGCATGAG
MYC67-5 (F)	GACAACTCTTGAAGATCAAAC
MYC67-SGR (R)	CAAGTGATATGATTCCTTTGAGAAG
MYC70-10 (F)	CAAAGTAGCAAACTCAAAATC
MYC70-SGR (R)	GAATCGAAGTTTCTCCAGCAATA
NOS-transR	GCCAAATGTTTGAACGATCGGGAA

Table S3. Primers for identification of T-DNA insertion line

Name of primer	DNA sequence (5'-to-3')
SALK009770L	GTTTCAAGGACACATCAACCC
SALK009770R	TGTATGATTTGCTCGAGGACC
SALK069605L	AAACTGCCACAGCCAATAATG
SALK069605R	TGGGTTGAATGGAAGTTCTTG
Salk_LBa1	TGGTTCACGTAGTGGGCCATCG

Table S4. Primers used for detection of the *CBF3/DREB1A* promoter

Name of primer	DNA sequence (5'-to-3')
CBF3-CHIP-aF	GTTCTATCGGACTAATTCTTGGC
CBF3-CHIP-aR	AAGAAAAAGAGTCTACGATGGATGA
CBF3-CHIP-bF	TAGGTTTCAGACTCGTTTCGC
CBF3-CHIP-bR	CTTTAGGAAAACCCTAAATAGACTGC
CBF3-CHIP-cF	TTAATTCAACCGTAAAAAGCAC
CBF3-CHIP-cR	TCTCCCATTCTTATAGCACATCC
CBF3-CHIP-dF	CAGATCTTAATGAGTGAATCCTTAAAC
CBF3-CHIP-dR	AATAAAAGTTGGAGTGAGAGCATGCTG
